# The Avian Diet Database as a source of quantitative information on bird diets

**DOI:** 10.1038/s41597-021-01049-9

**Published:** 2021-10-04

**Authors:** Allen H. Hurlbert, Aaron M. Olsen, Melissa M. Sawyer, Patrick M. Winner

**Affiliations:** 1 Department of Biology, University of North Carolina, Chapel Hill, NC 27599 USA; 2 Energy, Ecology and Environment Program, University of North Carolina, Chapel Hill, NC 27599 USA; 3 Department of Ecology and Evolutionary Biology, Brown University, Providence, RI 02912 USA; 4 Present Address: NOVA Engineering and Environmental, 2201 Brentwood Rd Suite 105, Raleigh, NC 27604 USA

**Keywords:** Food webs, Macroecology

## Abstract

This data paper describes a compilation of 73,075 quantitative diet data records for 759 primarily North American bird species, providing standardized information not just on the diet itself, but on the context for that diet information including the year, season, location, and habitat type of each study. The methods used for collecting and cleaning these data are described, and we present tools for summarizing and visualizing diet information by bird species or prey.

## Abstract (summary)

**Table:** 

Measurement(s)	Diet
Technology Type(s)	digital curation
Factor Type(s)	species
Sample Characteristic - Organism	Aves

Machine-accessible metadata file describing the reported data: 10.6084/m9.figshare.15088245

## Background & Summary

Diet is one of the most fundamental aspects of an organism’s ecology, and a knowledge of diet is required to better understand connections within food webs, potential determinants of population size and distribution, and potential conservation threats. For birds, existing compilations exist that classify diet broadly into categories such as insectivore, omnivore, and frugivore^1,2^, but finer resolution information about the specific prey items used and their relative importance are typically scattered throughout the literature in studies focused on only one or a few species at a time. One exception is the effort by the United States Department of Agriculture in the early twentieth century to quantify the stomach contents of over 100 North American bird species, identifying prey items to species where possible^3–10^. Nevertheless, these data are often reported in verbal summaries, and the taxonomy of both birds and prey items is in many cases outdated.

This data paper describes a compilation of 73,075 quantitative diet data records for 759 bird species, providing standardized information not just on the diet itself, but on the context for that diet information including the year, season, location, and habitat type of each study. While the species in this compilation are primarily from North America, this dataset will continue to grow and may expand in geographic coverage in the future. Below we describe our procedures for identifying datasets, standardizing those data, and conducting quality control and quality assurance checks.

## Methods

### Data discovery

We searched the literature for studies with quantitative data on diet. For North American bird species, we examined every citation we could access from the “Diet and Foraging” section of each relevant Birds of North America^11^ (now Birds of the World, birdsoftheworld.org) species account. In addition, we performed literature searches on Google Scholar and Web of Science using the keywords “[common name] AND (diet OR foraging OR food)”, replacing [common name] with the name of the bird species under investigation. For Anseriformes, studies were located using Google Scholar searches and Birds of North America species accounts, as described previously, in addition to ornithology handbooks^12–14^.

### Data entry

For each species for which quantitative diet data were found, we recorded details surrounding the study as described in Online-only Table 1. Researchers have historically used three different methods for quantifying the prevalence of a prey type. The fraction of the diet by number of items, the fraction of the diet by weight or volume, and the percent occurrence (i.e., the fraction of birds examined that consumed at least one member of a given a prey type). While the first two measures sum to 100% across all diet items in a study, the last one does not. In just a handful of studies, the authors quantified diet in terms of percent total energy content, or percent surface area of stomach contents examined on a miscroscope stage. For our standardization purposes, these were both classified as fraction of diet by weight or volume, but the exact method was recorded in the Notes field. In some cases, while quantitative information on diet was provided, the authors were unclear on which measure of prevalence was used, and we have recorded the Diet Type as “Unspecified”.

Some studies reported a non-zero, or “trace”, amount of a particular prey type in the diet, or reported an upper bound (e.g., “< 0.01”). In these cases, we entered a value of half of the lowest reported numeric diet fraction, or half of the upper bound. For example, if a study calculated that the prey item with the lowest representation in the diet that was still represented numerically was 0.014, and another prey item was reported as “trace”, that trace item would be entered as having a diet fraction of 0.007.

In some cases, researchers reported diet fractions for plant and animal portions of the diet separately (e.g., “beetles make up 30% of all animal food, grass seeds make up 10% of all plant food”). When possible, we used reported sample sizes of each diet portion to recalculate values that reflected the prevalence of those items in the diet as a whole.

### Bird taxonomy

Bird taxonomy in the database follows the 2019 Clements/eBird Checklist^15^. Species names as used in older published studies that did not match any names in the most recent Clements/eBird checklist at the time of data entry were searched in the online database AviBase (https://avibase.bsc-eoc.org/) which has features that allow one to map old names onto current taxonomic concepts. While simple name changes (e.g. genus *Dendroica* changing to *Setophaga*) were straightforward to incorporate, taxonomic splits were more complicated. For example, “Western Flycatcher” *Empidonax difficilis* was split into two species, Pacific-slope Flycatcher which retained the original scientific name, and Cordilleran Flycatcher, *Empidonax occidentalis*. Where possible, we used the geographic location of the study to determine which of the two split species was likely being referred to by the original study. When this was not possible due to range overlap of the two split species, the name would be reported as, for example, Pacific-slope/Cordilleran Flycatcher and *Empidonax difficilis/occidentalis*. In the case of taxonomic lumps, the relevant subspecies was listed for formerly distinct species when possible.

## Data Records

The latest official release of the database can be found in the file AvianDietDatabase.txt archived on Zenodo^16^, while the latest unofficial version to which new studies are continually added can be found in the AvianDietDatabase.txt file on Github (https://github.com/hurlbertlab/dietdatabase). Each record corresponds to a trophic link between one bird species and one prey taxon, along with the metadata detailing the location, date, habitat, and strength of this observation as characterized by the fields described in Online-only Table 1.

## Technical Validation

### Data quality checking

After initial data entry for a study, a cleaning script was run on the data to perform the following checks. 1) Bird common names, scientific names, and family names were checked against the 2019 Clements/eBird checklist^15^. 2) Data fields for which numeric values were expected were checked to ensure that values were indeed numeric, and potential outlier values were flagged based on accepted ranges of values for each field (Online-only Table 1). 3) Any values for Location_Region within North America that were not the names of U.S. or Mexican states or Canadian provinces were flagged, while outside of North America any values that were not country names were flagged. 4) Values for Habitat_type, Prey_Stage, Prey_Part, Diet_Type, and Study_Type were checked for allowable terminology (Online-only Table 1). 5) Finally, for each analysis (often a study, but occasionally a single study reported several diet analyses by season or habitat) broken down by number of items, weight or volume, or unspecified (but not by fraction occurrence), we checked that the Fraction_Diet values summed to 1. Analyses with sums that deviated by more than 0.03 (some small deviation is expected due to the summing of rounding errors) were compared to the original study for typos, and any records for which the analysis sum deviated from 1 by more than 0.03 have the phrase “values as reported do not sum to 100%” in the Notes field.

### Prey taxonomy and name cleaning

We recorded the names of prey items with as much taxonomic resolution as the original study allowed, following the Integrated Taxonomic Information System (ITIS; https://www.itis.gov) taxonomy where possible. If the reported prey name did not match any ITIS taxonomic concept, then it was entered into the Global Names Resolver (https://resolver.globalnames.org) to see whether it matched an outdated name that could be mapped to a current taxonomic concept in ITIS. We list the ITIS taxonomic serial number in the Prey_Name_ITIS_ID field and report the Prey_Name_Status as “verified” when a prey name was successfully matched to a concept in ITIS. Some names had no match in ITIS, but were regarded as valid taxonomic concepts by other authorities (e.g. Encyclopedia of Life, National Center for Biotechnology Information, World Register of Marine Species, etc.). In such cases, the Prey_Name_ITIS_ID was assigned ‘NA’ and we reported the Prey_Name_Status as “accepted”.

In some cases, researchers utilized an “other” or “unidentified” taxonomic category which required a particular form of encoding. For example, a study might report the diet fraction for “Ants”, “Vespid wasps”, and “Other wasps” or “Unidentified wasps”. These would be reported as “Hymenoptera: Formicidae”, “Hymenoptera: Vespidae”, and “Hymenoptera”, respectively. However, in this example, the last entry of “Hymenoptera” really refers to “all Hymenoptera not in Formicidae or Vespidae”, and is not comparable with the “Hymenoptera” of another study which was taxonomically inclusive. As such, we created a separate field in the database called “Prey_Taxon_Inclusive”. In this example, “yes” would be entered for both ants and vespid wasps because the diet fractions presented refer to the inclusive set of prey delimited by that prey name reported, but for the Hymenoptera record corresponding to “Unidentified wasps” the “Prey_Taxon_Inclusive” field would be “no”.

Taxonomic changes within the common insect order Hemiptera merit specific attention. Up until the late twentieth century, “Hemiptera” was used to refer to the True Bugs, which are now defined as the suborder Heteroptera within order Hemiptera. “Homoptera” had been used to refer to aphids, psyllids, leafhoppers, treehoppers and cicadas, but is now no longer considered a valid name. Instead, aphids, psyllids and scale insects make up the suborder Sternorrhyncha within Hemiptera, while leafhoppers, treehoppers, and cicadas make up the suborder Auchenorrhyncha within Hemiptera. Thus, an older study that reported prey as “Hemiptera” and “Homoptera” were entered as “Hemiptera: Heteroptera” and “Hemiptera”, with the latter listed as not inclusive. In some cases, studies provided additional information (e.g., “Homoptera (principally leafhoppers)”) allowing us to specify an inclusive suborder designation.

## Usage Notes

In addition to the raw data, we provide two means of exploring the Avian Diet Database and extracting species- or prey-specific summaries. The first is through the website https://aviandiet.unc.edu where users can enter a bird species name to explore a summary of diet information known for that species, or a prey name to explore which bird species are known to eat that prey taxon. We also provide an R package (‘aviandietdb’) for exploring the database, which should be loaded in R by typing:

install.packages("devtools")

library(devtools)

devtools::install_github("ahhurlbert/aviandietdb")

library(aviandietdb)

Three useful R functions for summarizing records in the database are detailed below.

*dbSummary()*.

Example usage:

dbSummary()

This function returns the total number of database records, the unique number of bird species, and the unique number of publications summarized in the Diet Database. In addition, it provides a tally of the number of records by bird species listed in alphabetical order, as well as a summary for each bird family in the American Birding Association (ABA) Checklist (version 8.0.6a) of 1) the number of species in the family in the database, 2) the total number of species in the family based on the ABA checklist, and 3) the percent of the family represented based on the species expected in North America. This information on taxonomic coverage is also provided in Online-only Table 2.

*speciesSummary()*.

Example usage:

speciesSummary("Bald Eagle", by = "Order")

This function provides a summary of the total number of records and total number of studies available in the database for this species, along with a summary of how those records are distributed across seasons, years, and geographic regions. The number of records are also summarized by taxonomic level to which prey were identified and by analysis type (by number of items, weight or volume, occurrence, or unspecified). Finally, for each analysis type, the mean fraction of diet is given for each prey category at the hierarchical taxonomic level specified with the “by” argument. This is an overall mean, averaged across year, region, and season. If the original data source indicated that specific parts of the prey taxon were consumed (e.g. fruit, seed, vegetation, etc.) then they are listed in the Prey_Part field.

*dietSummary()*.

Example usage:

dietSummary("Bald Eagle", season = "summer", region = "California", yearRange = c(1940, 1970), by = "Order", dietType = "Items")

This function allows one to specify season, region, a year range, analysis type, and taxonomic level for prey summarization, and then provides the mean fraction of diet information based on all studies meeting the stated criteria.

*dietSummaryByPrey()*.

Example usage:

dietSummaryByPrey("Lepidoptera", preyLevel = "Order", dietType = "Items", yearRange = c(1985, 2000), season = "summer", preyStage = "larva", speciesMean = TRUE)

This function provides a list of all bird species that consume a particular prey taxon in decreasing order of importance. In addition to providing the prey taxon name, you must also specify the taxonomic level (*preyLevel*) of that name. Like *dietSummary()*, this function allows one to specify season, region, a year range, and analysis type. There are two additional arguments not present in dietSummary(). One is *preyStage*, which specifies the life stage of the prey item (if applicable) for which a summary should be conducted. By default (‘any’), diet records will be included regardless of prey stage. Alternatively, one can specify that the summary should only be conducted for records including the terms ‘larva’, ‘adult’, or ‘pupa’ in the Diet Database’s ‘Prey_Stage’ field. This is most relevant for Lepidoptera and a few other insect groups, where one might want to single out the importance of caterpillars or other larvae, for example.

By specifying *speciesMean* = TRUE, only a single value is returned for each bird species that is known to consume a specified prey taxon which represents the average across all analyses meeting the season, region, and year criteria. If *speciesMean* is FALSE, then each analysis of a bird species which meets the specified criteria will be listed separately.

Example code and output is available in the Github README.md document (https://github.com/ahhurlbert/aviandietdb).

## Data Availability

R code for quality assurance/quality control and prey taxonomic name cleaning can be found in the “database_error_checking.R” script in the database building project’s Github repository (https://github.com/hurlbertlab/dietdatabase). An R package with functions for loading and querying the database is called ‘aviandietdb’ and is available at https://github.com/ahhurlbert/aviandietdb.

## Online-only Tables

**Online-only Table 1:** Database field names, their definition, data type, and range of acceptable values.

Field	Definition	Data type	Acceptable values
Common_Name	The common name of the species whose diet is being characterized, following the most recent Clements / eBird checklist.	character	
Scientific_Name	Genus and species of the species whose diet is being characterized, following the most recent Clements / eBird checklist.	character	
Family	Family of the species whose diet is being characterized.	character	
Taxonomy	The taxonomic authority being followed, currently eBird Clements Checklist v2018.	character	
Longitude_dd	Longitude of the study, if provided, in decimal degrees.	numeric	[−180, 180]
Latitude_dd	Latitude of the study, if provided, in decimal degrees.	numeric	[−90, 90]
Altitude_min_m	Minimum altitude of the study, if provided, in meters.	numeric	[−100, 10000]
Altitude_mean_m	Altitude of the study in meters if a single value was provided.	numeric	[−100, 10000]
Altitude_max_m	Maximum altitude of the study, if provided, in meters.	numeric	[−100, 10000]
Location_Region	Location of the study based on national or subnational place name (e.g., Florida, or Jamaica). If the study spans multiple regions they can be listed separated by semi-colons (e.g. “Florida; Alabama; Georgia”).	character	
Location_Specific	Location of the study using the most specific placename provided in the study (e.g. Hubbard Brook Experimental Forest, or Huachuca Mountains, or Dare County).	character	
Habitat_type	List one or more of the following habitat designations describing the habitat in which the study was conducted.	character	agriculture; broadleaf evergreen forest; coniferous forest; deciduous forest; desert; estuary; forest; grassland; ice/rock; lake; mangrove; marine; mudflats; river; rock; scrubland; tundra; urban; wetland; woodland
Observation_Month_Begin	The month in which diet data were first collected.	numeric	[1, 12]
Observation_Year_Begin	The year in which diet data were first collected.	numeric	[1800, 2020]
Observation_Month_End	The month in which diet data were last collected.	numeric	[1, 12]
Observation_Year_End	The year in which diet data were last collected.	numeric	[1800, 2020]
Observation_Season	The season(s) in which diet data were last collected. Two or more values may be listed separated by a semi-colon, e.g. “spring; summer”.	character	fall; multiple; spring; summer; winter
Analysis_Number	If multiple diet analyses were conducted for the same combination of location, habitat, season, and dates within the same study (e.g. if stomach contents for individual birds were reported separately), then each analysis is assigned a different integer incrementing from 1.	integer	[1, 20]
Prey_Kingdom	Kingdom to which the prey item belongs.	character	Animalia; Bacteria; Chromista; Fungi; Non-biological; Plantae; Protozoa; Unknown
Prey_Phylum	Phylum to which the prey item belongs.	character	
Prey_Class	Class to which the prey item belongs.	character	
Prey_Order	Order to which the prey item belongs.	character	
Prey_Suborder	Suborder to which the prey item belongs.	character	
Prey_Family	Family to which the prey item belongs.	character	
Prey_Genus	Genus to which the prey item belongs.	character	
Prey_Scientific_Name	The full scientific name, genus and species, to which the prey item belongs.	character	
Inclusive_Prey_Taxon	Does the % diet data listed correspond to the importance of the listed prey taxon inclusively defined (“yes”) or only for an unstated subset of the listed prey taxon (“no”) as in “Unidentified beetles”. See text for discussion.	character	no; yes
Prey_Name_ITIS_ID	The Integrated Taxonomic Information Service (ITIS) taxon ID associated with the prey item. This field should be left blank and will be populated by an R script automatically.	numeric	
Prey_Name_Status	Taxonomic status of the prey name, “verified” indicates the name matched a valid ITIS ID. “unverified” means the name did not match a valid ITIS ID and needs to be investigated further. “accepted” means the name did not match a valid ITIS ID, but investigation revealed that it reflects an accepted taxonomic entity not in ITIS.	character	accepted; unverified; verified
Prey_Stage	The lifestage of the identified prey item. In general, this is only specified if it is explicitly provided in the data source. For Lepidoptera only, ‘unspecified’ is used to make clear that the original source could be referring to larvae, pupae, or adults.	character	adult; egg; juvenile; larva; nymph; pupa; teneral; unspecified
Prey_Part	The part of the prey item represented in the diet if only a part was (likely) consumed; especially specified for plant-based diet items.	character	bark; bud; bulb; feces; flower; fruit; gall; nectar; oogonium; pollen; root; seed; spore; statoblasts; tuber; vegetation
Prey_Common_Name	Common name of the prey item if provided.	character	
Fraction_Diet	Fraction of the bird’s diet made up by this prey item.	numeric	[0, 1]
Diet_Type	The method by which Fraction_Diet was calculated. “Items”: Fraction of the diet as measured by a count of the number of prey items. E.g., the number of beetles in the stomach contents were counted, and this value was divided by the total number of unique prey items in the stomach contents. “Wt_or_Vol”: Fraction of the diet as measured by weight or volume. E.g., all beetles in the stomach contents were weighed, and this value was divided by the mass of all stomach contents. “Occurrence”: Fraction of the birds examined that contained at least one individual of this prey type. “Unspecified”: Fraction of the diet of the prey item based on a methodology unspecified by the authors.	character	Items; Occurrence; Unspecified; Wt_or_Vol
Item_Sample_Size	Total number of prey items identified in the diet sample.	numeric	[0, 64000]
Bird_Sample_Size	Total number of individuals of the focal bird species used to characterize diet.	numeric	[0, 4900]
Sites	Number of distinct study sites over which individuals were observed to characterize the diet.	numeric	[0, 100]
Study_Type	The way that diet data were collected.	character	behavioural observation; crop contents; emetic; esophagus contents; fecal contents; nest debris; pellet contents; prey remains; stomach contents
Notes	Any useful information about the nature of the study or the diet information that does not fit in the previous fields. If records in an analysis do not sum to between 0.97 and 1.03, then the following string will be included in the notes: “values as reported do not sum to 100%”	character	
Entered_By	Initials of the person entering the data.	character	
Source	Citation of the study from which the diet information comes.	character	

**Online-only Table 2:** Taxonomic coverage of the Avian Diet Database by family based on the total number of species in North America on the American Birding Association (ABA) checklist (https://www.aba.org/aba-checklist/).

Order	Family	Species with data	ABA total species	Percent coverage
Accipitriformes	Accipitridae	25	35	71
Accipitriformes	Pandionidae	1	1	100
Anseriformes	Anatidae	102	116	88
Bucerotiformes	Upupidae	0	1	0
Caprimulgiformes	Apodidae	2	10	20
Caprimulgiformes	Caprimulgidae	5	10	50
Caprimulgiformes	Trochilidae	16	34	47
Cathartiformes	Cathartidae	3	4	75
Charadriiformes	Alcidae	13	23	57
Charadriiformes	Charadriidae	1	17	6
Charadriiformes	Laridae	34	55	62
Charadriiformes	Recurvirostridae	1	3	33
Charadriiformes	Scolopacidae	17	66	26
Charadriiformes	Stercorariidae	4	5	80
Charadriiformes	Burhinidae	0	1	0
Charadriiformes	Glareolidae	0	1	0
Charadriiformes	Haematopodidae	0	3	0
Charadriiformes	Jacanidae	0	1	0
Ciconiiformes	Ciconiidae	1	2	50
Columbiformes	Columbidae	12	20	60
Coraciiformes	Alcedinidae	0	4	0
Cuculiformes	Cuculidae	6	12	50
Falconiformes	Falconidae	6	11	55
Galliformes	Cracidae	7	7	100
Galliformes	Odontophoridae	6	6	100
Galliformes	Phasianidae	16	23	70
Gaviiformes	Gaviidae	4	5	80
Gruiformes	Aramidae	1	1	100
Gruiformes	Gruidae	1	3	33
Gruiformes	Rallidae	8	20	40
Gruiformes	Heliornithidae	0	1	0
Passeriformes	Aegithalidae	1	1	100
Passeriformes	Alaudidae	1	2	50
Passeriformes	Bombycillidae	1	2	50
Passeriformes	Calcariidae	5	6	83
Passeriformes	Cardinalidae	13	19	68
Passeriformes	Certhiidae	1	1	100
Passeriformes	Corvidae	18	24	75
Passeriformes	Estrildidae	2	8	25
Passeriformes	Fringillidae	17	71	24
Passeriformes	Hirundinidae	9	16	56
Passeriformes	Icteridae	19	30	63
Passeriformes	Icteriidae	1	1	100
Passeriformes	Laniidae	2	4	50
Passeriformes	Mimidae	7	13	54
Passeriformes	Motacillidae	2	10	20
Passeriformes	Muscicapidae	1	18	6
Passeriformes	Paridae	7	12	58
Passeriformes	Parulidae	44	58	76
Passeriformes	Passerellidae	31	44	70
Passeriformes	Passeridae	1	2	50
Passeriformes	Polioptilidae	2	4	50
Passeriformes	Ptiliogonatidae	1	2	50
Passeriformes	Regulidae	2	2	100
Passeriformes	Remizidae	1	1	100
Passeriformes	Sittidae	4	4	100
Passeriformes	Spindalidae	1	2	50
Passeriformes	Sturnidae	1	2	50
Passeriformes	Sylviidae	1	2	50
Passeriformes	Thraupidae	5	11	45
Passeriformes	Tityridae	1	4	25
Passeriformes	Troglodytidae	9	12	75
Passeriformes	Turdidae	13	33	39
Passeriformes	Tyrannidae	36	54	67
Passeriformes	Vireonidae	12	18	67
Passeriformes	Acrocephalidae	0	4	0
Passeriformes	Cinclidae	0	1	0
Passeriformes	Emberizidae	0	9	0
Passeriformes	Leiothrichidae	0	3	0
Passeriformes	Locustellidae	0	3	0
Passeriformes	Mohoidae	0	5	0
Passeriformes	Monarchidae	0	3	0
Passeriformes	Peucedramidae	0	1	0
Passeriformes	Phylloscopidae	0	8	0
Passeriformes	Prunellidae	0	1	0
Passeriformes	Pycnonotidae	0	2	0
Passeriformes	Scotocercidae	0	1	0
Passeriformes	Zosteropidae	0	1	0
Pelecaniformes	Ardeidae	17	24	71
Pelecaniformes	Pelecanidae	3	3	100
Pelecaniformes	Threskiornithidae	4	5	80
Phaethontiformes	Phaethontidae	3	3	100
Phoenicopteriformes	Phoenicopteridae	0	1	0
Piciformes	Picidae	20	26	77
Podicipediformes	Podicipedidae	8	8	100
Procellariiformes	Diomedeidae	7	12	58
Procellariiformes	Hydrobatidae	4	12	33
Procellariiformes	Oceanitidae	2	3	67
Procellariiformes	Procellariidae	39	61	64
Psittaciformes	Psittacidae	3	9	33
Psittaciformes	Psittaculidae	1	3	33
Pterocliformes	Pteroclidae	0	1	0
Strigiformes	Strigidae	19	23	83
Strigiformes	Tytonidae	1	1	100
Suliformes	Anhingidae	2	2	100
Suliformes	Fregatidae	3	3	100
Suliformes	Phalacrocoracidae	9	9	100
Suliformes	Sulidae	6	7	86
Trogoniformes	Trogonidae	2	3	67

In addition, the database includes diet data for 36 other species not on the ABA checklist.
